# ARRIGE Arrives: Toward the Responsible Use of Genome Editing

**DOI:** 10.1089/crispr.2018.29012.mon

**Published:** 2018-04-01

**Authors:** Lluis Montoliu, Jennifer Merchant, François Hirsch, Marion Abecassis, Pierre Jouannet, Bernard Baertschi, Cyril Sarrauste de Menthière, Hervé Chneiweiss

**Affiliations:** ^1^ National Centre for Biotechnology (CNB-CSIC), CSIC Ethics Committee and Biomedical Research Networking Centre Consortium on Rare Diseases (CIBERER-ISCIII), Madrid, Spain.; ^2^ University Panthéon-Assas, Paris, France.; ^3^ French National Institute for Health and Medical Research (INSERM) Ethics Committee, France.; ^4^ University Paris Descartes, Paris, France.; ^5^ University of Geneva, Geneva, Switzerland.; ^6^ Institute of Human Genetics (IGH), National Center for Scientific Research (CNRS), University of Montpellier, Montpellier, France.; ^7^ CNRS UMR8246, INSERM U1130 and University Pierre and Marie Curie UMCR18, Neuroscience Paris Seine-IBPS, Team Glial Plasticity, Paris, France.

## In March 2018, Approximately 160 Participants from 35 Countries Gathered in Paris to Launch the ARRIGE (Association for Responsible Research and Innovation in Genome Editing) Initiative

Genome editing is a transformative technology that allows precise and sophisticated genetic alterations in any genome thanks to a variety of molecular editors. The CRISPR^*^-Cas9 genome-editing technology, derived from prokaryotic adaptive immune systems, has transformed targeted editing into a practical reality, widely available and affordable.^1^ Numerous applications have already been explored in model systems, animals, and plants for biological and biotechnological purposes, improving the production and nutritional value of food, and/or improving adaption to an environment. However, biomedical applications—the great hope for treating and potentially curing many genetic diseases—have yet to be effectively deployed, requiring the careful evaluation of safety and efficacy constraints before entering the clinic.

Despite the countless potential benefits of CRISPR-Cas genome editing, farmers, patients, and many other citizen groups are largely unaware of the effects, risks, and profound implications associated with the heritable modification of organismal genomes, including the human genome. Common controversies seem to be associated with nonmedical applications, whereas medical uses of new technologies normally have a broader acceptance in society. Traditional communication schemes used by academic and private researchers of late for sharing the benefits of, for example, transgenic plants, have fallen short or proven counterproductive. In contrast, the adoption of openness and transparency initiatives has reaped enormous benefits for the understanding and acceptance of required experimentation with laboratory animals.^2^ The main difference is complete clarity and direct communication with key stakeholders, providing information about what, where, for which purposes, who, and how many animals are to be used in the proposed research. Raised awareness is a key first step to allow public stakeholders to debate and judge the uncertainties and transformative potential of genome-editing technologies.

## The French Connection

In 2015 a group of researchers and ethicists from France and neighboring countries, initiated by the National Institute for Health and Medical Research (INSERM) Ethics Committee, began organizing a series of meetings in Europe, India, Africa, and South America, addressing the diverse ethical issues associated with the responsible use of genome editing as applied to humans, animals, plants, and the environment. The initial position of this group, projected from Europe, was first reported in early 2017.^3^ The proposal was expanded upon in a longer paper published last summer that proposed the creation of a European Steering Committee to assess the potential benefits and risks of genome editing, design risk matrices and scenarios for responsible uses of this technology, and contribute to an open debate on societal aspects prior to a translation into national and international legislation.^4^

In November 2017, INSERM held an important meeting in Paris to examine many existing reports, position papers, and manifests on the ethical and societal aspects of genome editing and the responsible uses of these technologies (reviewed by de Lecuona *et al*.).^5^ We wanted to go beyond the publication of a simple report: we wished to become useful, operative, proactively engaging the various stakeholders mentioned above in this debate.

**FIG. 1.**
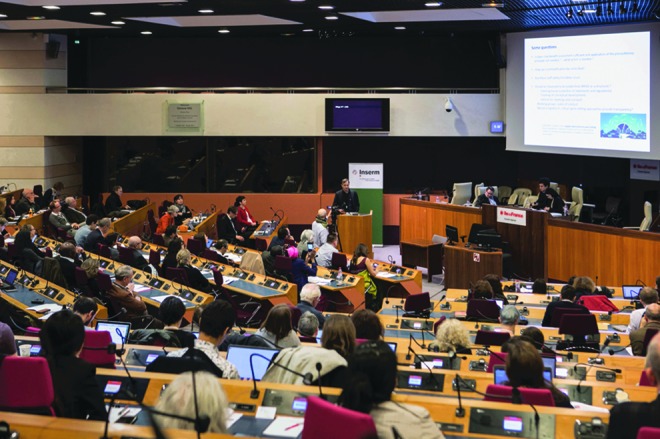
ARRIGE kick-off meeting at the Region Île-de-France Parliament in Paris on March 23, 2018. (Photograph courtesy of INSERM.)

From that meeting, it also became clear that the scope of this initiative had to be truly international, going beyond the usual perspectives from Europe, North America, China, Japan, and Australia and involving the oft-forgotten south by including members from Southeast Asia, Africa, and Central/South America. Those discussions crystalized in the March 23, 2018, conference at Île-de-France regional Parliament in Paris, featuring approximately 160 participants from 35 countries. Here we decided to launch the ARRIGE initiative (Fig. 1).

The aim of this new nonprofit initiative is to promote a global governance of genome editing through a comprehensive setting for all stakeholders—academics, researchers, clinicians, public institutions, private companies, patient organizations, and other nongovernmental organizations, regulators, citizens, media, governmental agencies, and decision makers from all continents. We hope to address multiple issues raised by genome-editing technologies used in research and applications within a safe and ethical framework for individuals and society.

More specifically, the ARRIGE association aims to provide a vehicle for meetings and outreach with the following four major objectives:

(1) fostering an inclusive debate with a risk-management approach, considering human, environmental, animal, and economic issues;
(2) getting involved in the governance of genome-editing technology with governmental and intergovernmental stakeholders;
(3) creating an ethical toolbox and informal guidance for genome-editing technology users, regulators, governance, and the civil society at large, including those living in low- and middle-income countries; and
(4) developing a robust and specific reflection on the role of the lay public in this debate and the necessity for improved public engagement.

Coincidentally, the same week of our March meeting in Paris, a similar proposal was published in *Nature*, suggesting the creation of a global observatory and requesting a cosmopolitan conversation on the uses, applications, and consequences of genome editing technologies.^6^ These proposals were independent of each other. However, thanks to media outreach,^7^ we look forward to working collaboratively in pursuit of our common aims and interests.^*^

## Notes

^*^ ***C****lustered* ***R****egularly* ***I****nterspaced* ***S****hort* ***P****alindromic* ***R****epeats.*

^*^ For more information, contact the ARRIGE initiative (join@arrige.org) and/or visit the ARRIGE web site, which hosts various documents, talks, and videos (http://arrige.org).

## References

[1] Mojica FJM, Montoliu L On the Origin of CRISPR-Cas technology: from prokaryotes to mammals. Trends Microbiol. 2016;24:811–820. DOI: 10.1016/j.tim.2016.06.005 27401123

[2] Jarrett W The Concordat on openness and its benefits to animal research. Lab Anim (NY). 2016;45:201–202. DOI: 10.1038/laban.1026 27203253

[3] Hirsch F, Lévy Y, Chneiweiss H CRISPR-Cas9: A European position on genome editing. Nature. 2017;541:3 0. DOI: 10.1038/541030c 28054606

[4] Chneiweiss H, Hirsch F, Montoliu L, et al. Fostering responsible research with genome editing technologies: a European perspective. Transgenic Res. 2017;26:709–713. DOI: 10.1007/s11248-017-0028-z 28730514 PMC5601998

[5] de Lecuona I, Casado M, Marfany G, et al. Gene Editing in humans: towards a global and inclusive debate for responsible research. Yale J Biol Med. 2017;90:673–681. eCollection 2017 Dec 29259532 PMC5733855

[6] Jasanoff S, Hurlbut JB A global observatory for gene editing. Nature. 2018;555:435–437. DOI: 10.1038/d41586-018-03270-w 32034373

[7] Enserink M Interested in responsible gene editing? Join the (new) club. Science News, March 27, 2018 www.sciencemag.org/news/2018/03/interested-responsible-gene-editing-join-new-club (last accessed 44, 2018)

